# Patient and provider attitudes to emergency department-based HIV counselling and testing in South Africa

**DOI:** 10.4102/sajhivmed.v18i1.707

**Published:** 2017-05-31

**Authors:** Bhakti Hansoti, Sarah E. Hill, Madeleine Whalen, David Stead, Andy Parrish, Richard Rothman, Yu-Hsiang Hsieh, Thomas C. Quinn

**Affiliations:** 1 Department of Emergency Medicine, Johns Hopkins University, United States; 2 Kriegler School of Arts and Sciences, Johns Hopkins University, United States; 3 Department of Internal Medicine, Frere Hospital, South Africa; 4 Department of Medicine, Walter Sisulu University, South Africa; 5 Division of Intramural Research, NIAID, NIH, Bethesda, United States

## Abstract

**Background:**

The national South African HIV Counselling and Testing (HCT) guidelines mandate that voluntary counselling and testing (VCT) should be offered in all healthcare facilities. Emergency departments (EDs) are at the forefront of many healthcare facilities, yet VCT is not routinely implemented in this setting.

**Methods:**

We conducted a cross-sectional study that surveyed patients and healthcare providers at a tertiary care ED in the spring and summer of 2016 to ascertain their attitudes to VCT in the ED. We also used two previously validated survey instruments to gather data on patients’ HIV knowledge and providers’ stigma against patients living with HIV, as we anticipated that these may have an impact on providers’ and patients’ attitudes to the provision of HIV testing within the ED, and may offer insights for future intervention development.

**Results:**

A total of 104 patients and 26 providers were enrolled in the study. Overall, patients responded more favourably to ED-based HIV testing (92.3%) compared to providers (only 40% responded favourably). When asked about potential barriers to receiving or providing HIV testing, 16.4% of patients and 24% of providers felt that the subject of HIV was too sensitive and 58.7% of patients and 80% of providers indicated that privacy and confidentiality issues would pose major barriers to implementing ED-based HIV testing.

**Conclusion:**

This study shows that while ED-based HIV testing is overall highly acceptable to patients, providers seem less willing to provide this service. The survey data also suggest that future development of ED-based testing strategies should take into consideration privacy and confidentiality concerns that may arise within a busy emergency care setting. Furthermore, every effort should be made to tackle HIV stigma among providers to improve overall attitudes towards HIV-positive individuals that present for care in the ED.

## Introduction

HIV infection is a worldwide public health problem that disproportionately affects vulnerable population groups and populations in low-resource settings. In sub-Saharan Africa, nearly one in every 20 adults is living with HIV, and over 25% of those infected remain unaware of their HIV-positive serostatus.^1^ This is despite ongoing efforts to bring screening to the general population.^1^ To address the testing gap, and in accordance with recommendations from the World Health Organization (WHO), the South African Department of Health and the United States Centers for Disease Control and Prevention (CDC), public health leaders in South Africa have begun to recognise the importance of transitioning HIV testing and linkage to care from traditional outpatient settings, to other components of the healthcare system including high-impact, population rich sectors such as the nation’s emergency departments (EDs).^2^

In 2010, the South African Department of Health released explicit requirements that provider-initiated counselling and testing (PICT) be offered in all healthcare facilities, including emergency care. The relative lack of trained HIV counsellors, coupled with already overburdened clinical resources in settings such as EDs, has significantly impeded uptake, such that testing services currently remain focused in community-based clinics, mobile clinics and antenatal centers.^3^ Significant challenges thus remain, to addressing the Joint United Nations Program on HIV/AIDS (UNAIDS) target, namely that 90% of HIV-positive individuals be diagnosed by 2020. For example, a 2015 South African study found that rates of HIV testing remain exceedingly low among men and older adults.^4^

In many countries, EDs are considered the safety net of the healthcare system since they are required to provide care to all patients with acute and life threatening injury and illness. In South Africa, emergency care is enshrined in the constitution as a basic human right^2^ assuring delivery of care to large volumes of patients. In high-resource settings, such as the United States, EDs have proven to be pivotal both for defining the burden of HIV, and developing high yield programmatic testing and linkage to care.^5,6^

Pilot studies from other sub-Saharan countries have also identified the ED to be a high yield testing venue.^7,8^ In Uganda, Nakanjako et al.^9^ reported an HIV prevalence among ED patients of 30%. It was also reported that 99% of patients believed HIV testing and counselling should be a part of routine care in the hospital setting.^9^ Based on these results, HIV testing in the ED appears to be a promising and highly acceptable testing intervention.

South Africa has the largest HIV epidemic in the world.^10^ The Eastern Cape region of South Africa, in particular, faces a disproportionate burden of HIV infection.^11^ ED-based testing may be an important setting to help close testing coverage gaps, both in terms of number of patients reached as well as reaching those who may not get tested elsewhere, for example, young men who do not seek routine healthcare.^12^ The implementation of any new service intervention within an already complex healthcare setting requires an assessment of both acceptability and feasibility. This study forms part of a larger research strategy that focuses on the development of an ED-based HIV testing and linkage to care for the South African emergency care setting. The first component of this evaluation was to gauge local acceptability of ED-based HIV testing to inform intervention implementation. Future studies will conduct exploratory evaluations to identify implementation gaps and assess intervention feasibility. In this study, we assessed patient and provider attitudes towards ED-based HIV testing. We also surveyed patients’ knowledge of HIV and providers’ stigma against patients living with HIV, as we hypothesised that these factors may influence patient and healthcare provider attitudes to provision of HIV testing within the ED.

## Methods

### Study design

We designed a cross-sectional, observational study that surveyed patients and healthcare providers at a tertiary care ED in the spring and summer of 2016.

### Study setting

The study was conducted in the ED of Frere Hospital located in East London, South Africa. Frere Hospital is a provincial, government funded hospital located in the Eastern Cape in South Africa. The Frere Hospital ED has 24 hour emergency care coverage, and provides access to all public sector patients in the East London region that present with medical or surgical emergencies. The Frere Hospital ED sees approximately 120–150 patients per day, at any time there are two physicians, six nurses and six nursing assistants to care for patients in the ED. The average nurse to patient ratio can be as high as 1–10 in certain parts of the ED.

### Enrolment and eligibility

A convenience sample of patients and providers was recruited for voluntary and anonymous participation using a verbal consent script. Only patients who were over the age of 18 years and spoke English were approached for enrolment. Recruitment took place in the waiting room while patients waited for a provider after triage was completed so as not interfere with patient care. An announcement was made to the entire clinical staff team every morning during their routine hand-off session. Providers who wished to participate were asked to liaise with a member of the research team during their break period.

### Data collection instruments and strategy

Following informed consent, patients were asked to complete the Patient Attitudes to HIV Testing (43 questions) and patient knowledge of HIV questionnaires (11 questions), as well as basic demographic details. Providers were requested to complete the Healthcare Provider HIV/AIDS Stigma Scale (HPASS) questionnaire (30 questions) and a Staff Attitudes to HIV Testing in the ED survey (10 questions) as well as provide demographic details. The HPASS questionnaire and the patient knowledge questionnaire have been previously validated.^13,14^ Given the lack of a previously validated survey instrument, the ED attitudes to HIV testing surveys were created by the study team using currently available peer-review published instruments.^15,16,17,18,19,20^

The study team collected the data using an electronic handheld mobile tablet. Each survey contained a brief introduction and a consent script. Completion of the survey served as the official record that verbal consent to participate in the study had been obtained. Patients and providers were asked each question and then allowed to read it before providing an answer. Interviews took place in a private setting to ensure confidentiality for survey administration, and no patient or provider-identifying information was collected.

### Outcome measures and data analysis

The primary outcome measure was patient and staff acceptability of an ED-based HIV testing strategy. The secondary outcome measures were patient knowledge of HIV infection and staff stigma towards providing care for patients living with HIV. Simple descriptive statistics were derived with Microsoft Excel (Microsoft, Redmond, WA) and STATA^©^ (Stata Corporation, College Station, TX). Data analysis was conducted using cross tabulation. The Likert scale responses were collected as ‘strongly agree’, ‘agree’, ‘neither agree nor disagree’, ‘disagree’ and ‘strongly disagree’. A *p*-value less than 0.05 was considered statistically significant.

## Results

### Demographics

A total of 104 patients and 26 providers were enrolled in the study (Table 1). Basic demographic information from both groups are summarised in Table 1. The majority of patients were over the age of 30 years, unemployed, single and had received less than a high school diploma. Of the providers who were interviewed, 64% were nurses and 32% were doctors and the remaining 4% identified as ‘other’ (which included hospital administrators, nursing students and clerical staff). The majority of those interviewed were women, had been in practice for over 5 years (88%) and worked in Frere Hospital for 5–9 years (56%).

**TABLE 1:** Distribution of demographic characteristics of patients and providers in Frere Hospital, East London, South Africa.

Variable	Patients (*n* = 104)	Providers (*n* = 26)
Sex (male)	47.1%	16%
Age (greater than 30 years old)	67.3%	87.5%
Education (less than high school diploma)	69.2%	0%
Marital status (married)	35.6%	N/A
Employed	37.5%	100%
Average number of HIV tests since the age of 18	8.2	N/A

Overall, patients responded favourably to ED-based HIV testing (92.3%). However, this attitude was not mirrored by staff with only 40% agreeing with the statement ‘The emergency department should offer HIV testing’. Both patients and providers agreed that if testing was implemented, an HIV counsellor would be an acceptable person to deliver the results of an HIV test to a patient. When asked about potential barriers to HIV testing, 16.4% of patients and 24% of providers felt that the subject of HIV was too sensitive and 58.7% of patients and 80% of providers agreed that privacy and confidentiality issues were major barriers to implementing ED-based HIV testing (Table 2).

**TABLE 2:** Patient and provider response to key questions regarding emergency department-based testing strategy.

Statement	Patients (% agree)	Providers (% agree)
The emergency department should offer HIV testing.	92.3	40
An HIV counsellor would be an acceptable person to deliver the results of an HIV test.	72.1	92
The subject matter is too sensitive to conduct HIV testing in emergency departments.	16.4	24
Confidentiality/privacy concerns are a barrier to HIV testing in emergency departments.	58.7	80

**FIGURE 1:**
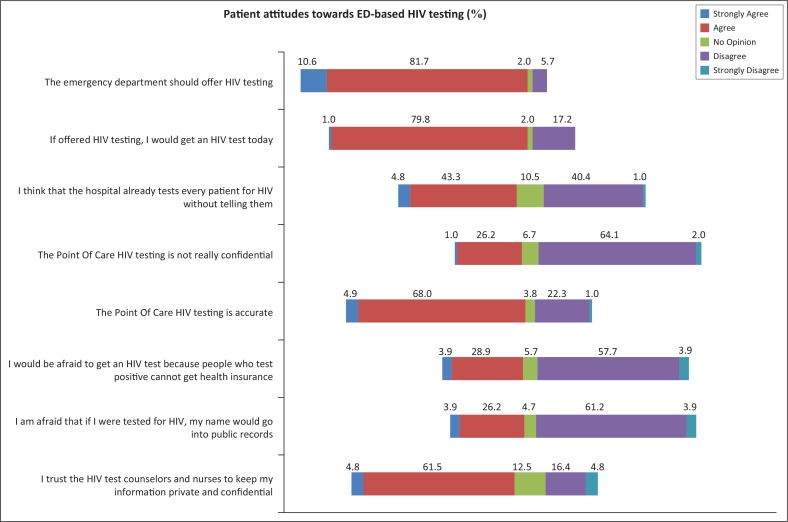
A graphical representation of key patient attitudes towards emergency departments-based HIV testing on a Likert scale.

The majority of patients agreed that the ED should offer HIV testing, and 78.2% agreed that if offered HIV testing, they would prefer to be tested that day (Figure 1). Patients strongly agreed that HIV testing should be free. Almost half of patients (48%) assumed that the hospital already tests every patient for HIV without telling them. Patients’ response to the survey indicated that they believed point of care HIV testing is both confidential and accurate (69.5%). Fifty one per cent of patients stated that they would not want anyone to know if they decided to be tested for HIV and only half of patients reported that they would trust nurses and HIV counsellors to keep their information private and confidential (Figure 1).

The most commonly cited barriers to implementation of routine ED-based HIV testing included privacy and confidentiality concerns (58.7%), that they already knew their HIV status (47.1%) and that this was not the primary purpose of their ED visit (41.3%). While 66.7% of patients would recommend a friend to get an HIV test in the ED, 80.8% of patients reported that they would not be willing to pay for an HIV test.

### Patient knowledge of HIV

In general, patient knowledge of HIV varied. When answering general knowledge questions about HIV, 99% of patients answered correctly regarding condom use for preventing HIV and 98% responded correctly that HIV causes AIDS. Patients had less information on vertical transmission (73.7% answered correctly) and asymptomatic HIV infection (73.8% answered correctly).

Throughout the study, relevant quotes from patients regarding perceptions of HIV testing and stigma towards HIV, were collected. The majority of statements (89%) collected centred around HIV stigma and HIV testing. Box 1 provides representative examples of these statements.

BOX 1 Patient quotes collected during interviews.

- Regarding HIV infection, ‘The stigma [towards HIV] is so bad here in South Africa. If I found out I was HIV positive, I would probably hang myself’.
- On privacy and confidentiality, ‘I am worried about the nurses and doctors revealing my [HIV] results to other patients. I don’t trust them’.
- Regarding stigma: ‘I think it is extremely important for everyone to know their HIV status, but I know many people don’t want to. They are afraid of people finding out, of what people will say about them. For example, some people may not let their children play with other children who have HIV’.
- On HIV testing, ‘South Africans are very judgmental; they would assume you had HIV even if you just got tested’.

### Impact of HIV knowledge towards testing

Patients’ level of HIV knowledge (poor knowledge was defined as getting less than 7 out of the 10 validated knowledge questions wrong) had no impact on their attitudes towards ED-based HIV testing. The mean knowledge score was 7.6 of 9 correct (range: 5–9), or 84% (SD: 15%). There was no statistical difference in mean HIV knowledge score between patients who reported strongly agree or agree opinion on the statement ‘The A&E should offer HIV testing’ and patients who reported neutral or disagree opinion (7.6 vs. 7.3 or 84% vs. 81%, *p* = 0.503).

### Provider attitudes to emergency department-based HIV testing

**FIGURE 2:**
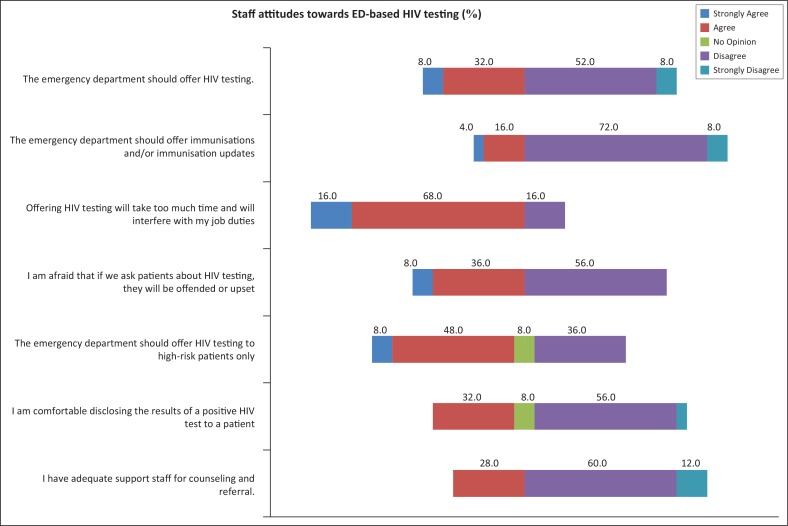
A graphical representation of key provider attitudes towards emergency department-based HIV testing on a Likert scale.

Overall, providers did not respond favourably to implementing an ED-based HIV testing strategy, with 80% disagreeing with the statement that the ED should offer HIV testing (Figure 2). Most (84%) providers agreed that HIV testing would take up too much time and interfere with their job duties. Nearly three quarter (72%) of providers disagreed with the statement that they believed they would have adequate support staff for delivery of counselling and referral. Only 32% of providers reported being comfortable disclosing the results of a positive HIV test to a patient; however, 56% of providers disagreed with the statement that patients would be offended or upset when offered an HIV test (Figure 2).

### Staff stigma towards caring for patients with HIV

In general, providers reported varying degrees of stigma. Most (76%) providers agreed that most HIV-positive patients acquired the virus through ‘risky’ behaviour, and 54.2% of providers agreed that people would not have HIV if they had sex with fewer people. When it came to working with colleagues who were HIV-positive, 68% of providers reported feeling comfortable working alongside another healthcare provider who had HIV. While 41.7% of providers would avoid conducting certain procedures on HIV-positive patients, only 28% agreed that they have the right to refuse to treat HIV-positive patients if it made them feel uncomfortable. In addition, 72% of providers worry about contracting HIV from HIV-positive patients.

Providers had many concerns about the implementation of an ED-based HIV testing strategy. Box 2 provides representative quotes collected from providers during the interview. Among providers, 83% of the responses were unfavourable towards an ED-based HIV testing strategy.

BOX 2 Quotes from providers collected during interview.

- ‘There are no private rooms for HIV counseling and testing. Everything is open; there is no space. This is a huge barrier of providing HIV testing in the ED’.
- ‘We [the nurses] have no time to do HIV testing. We are an emergency clinic, we deal with emergencies, not HIV’.
- On HIV stigma, ‘HIV is like another disease and I don’t like how much attention is placed towards it. We have been talking about it for too long for it to be a big deal’.

## Discussion

Our survey found that patients and providers vary markedly in their attitudes regarding provision of HIV testing and counselling in the ED. This is surprising given the fact that despite having the highest prevalence of HIV infection, South Africa also hosts the largest number of programmes, and most advanced infrastructure of any African country to enable linkage to care and treatment for HIV-infected individuals.^21^ In addition, our study found that many of the traditional barriers to HIV testing (e.g. concerns regarding confidentiality, accuracy of results and stigma in society) still prevail, particularly among providers, suggesting that policymakers and public health professionals must face significant hurdles to address patient and provider attitudes and concerns prior to implementing an ED-based HIV testing strategy.

Stigma not only affects healthcare providers’ provision of HIV testing, but also a patient’s willingness to accept an HIV test. The impact of stigma on HIV testing acceptance is not new. In a 2003 South African study, Kalichman et al. found that when compared with people who had been tested for HIV, individuals who had never been tested for HIV demonstrated greater HIV-related discrimination, ascribing greater guilt, shame and social disapproval to those living with HIV.^22^ In a study in Botswana two years before the implementation of universal access to HIV care, the majority of patients who delayed testing did so because of fear of HIV stigma.^23^ Notably, HIV-related stigma is not limited to developing countries.^24^ While efforts to reduce stigma have resulted in substantial gains,^25,26^ concerns regarding stigma persist worldwide and remain one of the most frequently cited barriers to HIV testing and treatment.^27,28^ In both developed and developing countries, research points to community-based education, as well as increased access to antiretroviral therapy (ART,) in reducing HIV stigma among the general community.^29,30,31^ The results of this study show that significant education efforts will be required to reduce HIV stigma among both healthcare providers and patients.

It has been six years since the South African National HIV Counselling and Testing (HCT) guidelines mandated that PICT should be offered to all persons attending medical services in both public and private sectors. Compliance remains poor because of the significant barriers (such as lack of trained HIV counsellors, time and cost of testing).^32^ In addition, PICT is time consuming and requires availability of trained full-time HIV counsellors to initiate testing, especially difficult in evenings and weekends, times when the majority of patients present for care in the ED.^33,34^ An alternative strategy may be to perform routine opt-out blood-based laboratory testing, for all patients who have blood drawn for clinical indications. The practical aspects of this concept require less ancillary staff to undertake pre-test counselling, while potentially de-stigmatising the disease by integrating it into the provision of routine care.^6^

A further complicating factor is that EDs are the busiest and most stressful units in hospitals.^6,35^ In our study, 84% of providers reported that HIV testing would take up too much time and interfere with their job duties. This statement may reflect an overall trend of burnout and job dissatisfaction among emergency service providers which is attributable to a variety of systemic factors, including difficult work schedules and lack of resources.^36,37^ The connection between HIV stigma and job dissatisfaction has been researched in many low-middle-income countries. Notably, one recent study in five African countries (Lesotho, Malawi, South Africa, Swaziland and Tanzania) indicated that perceived stigma against HIV negatively correlates with job dissatisfaction among nurses caring for HIV-positive individuals.^38^ Studies have found that HIV stigma experienced by nurses is a key contributing factor to their decision to immigrate and may contribute to health workforce shortages.^39,40^

This study suggests that HIV stigma is not only hindering timely HIV testing and diagnosis in the ED, but may also be a detrimental force in the maintenance of our valuable and finite emergency healthcare workers. To address HIV stigma and discrimination, it has been shown that universal testing and treatment should be integrated into the national response to HIV.^25^ EDs in the United States have made an effort to integrate opt-out HIV testing strategies into routine care to improve HIV testing uptake and de-stigmatise the provision of HIV testing.^41,42^ In South Africa, however, universal population-based opt-out testing will be challenging because the national standards dictate that patients must be provided with extensive testing prior to the offering of testing. Although streamlined population-based opt-out testing has the potential to reduce HIV testing stigma and increase testing rates in South Africa, pragmatic issues need to be addressed to capitalise on the benefits of this strategy. Barriers to these efforts include existing counselling standards, staff and patient HIV stigma, and the establishment of effective and reliable linkages to care programmes.^43^

### Limitations

A closed question survey instrument that has been previously used in the ED HIV literature was utilised in this study to gather information on patients and provider attitudes to HIV testing. The limitations of closed question survey are that while it enables you to get an overall gist of the current environment and is easily replicable across studies and sites, it does not allow one to explore the underlying reasoning behind the answers to survey questions. In our methodology, we did collect some free-text responses; however, an in-depth qualitative interview methodology will be necessary to explore the presented findings further. In addition, while our study demonstrated that providers in our sample had an overall negative attitude to ED-based HIV testing and there is some provider stigma against people living with HIV, we are unable to prove causation. Lastly, the study addresses only one aspect of pre-implementation evaluation; we do not address structural or organisational barriers to the provision of HIV testing and linkage to care within the emergency care context.

## Conclusion

Emergency department-based HIV testing is rarely implemented in South Africa. In this exploratory study, we have identified that while ED-based HIV testing is highly acceptable to patients, providers seem less willing to provide this service. Prior to implementing an ED-based testing strategy, it will be necessary to first address provider concerns surrounding resource utilisation, comfort and privacy for testing provision. Furthermore, to our surprise, our study sample raises some concerns about provider stigma against HIV-positive patients that warrants further exploration.
